# The Influence of Contextual Cues in Judgment Formation: An Ecologically Valid Test

**DOI:** 10.1371/journal.pone.0154383

**Published:** 2016-04-22

**Authors:** Jacob Jacoby, Jeff Galak

**Affiliations:** 1 Stern School of Business, New York University, New York, New York, United States of America; 2 Tepper School of Business, Carnegie Mellon University, Pittsburgh, Pennsylvania, United States of America; Center for BrainHealth, University of Texas at Dallas, UNITED STATES

## Abstract

An ecologically valid experiment investigated the propositions that (a) people’s judgments are influenced by contextual cues, (b) that they are often unaware that those cues influenced them, and (c) that even when they know the cues *should* influence them, they do not readily incorporate those cues into their judgment formation. After participating in a realistic simulation of a shopping experience, 405 consumers made judgments about whether the product they examined contained fresh or preserved grapefruit sections. Our findings show that despite being aware that contextual cues (such as the location within a store where the product is sold, the type of container it is sold in, and whether the container is chilled or not) generally influence the judgment at hand, people generally fail to realize that their specific judgments were influenced at all. These findings replicate prior studies, thereby extending the generalizability and robustness of prior research.

## Introduction

Nearly 40 years ago, William McGuire wrote:

We have mentioned several times in this discussion of the information processing steps in decision making that the person is often unconscious of what he or she is doing and, when explicitly questioned, is unable to give an adequate explanation of how the information was handled or the decision was reached. At other times, the person can report how the decision was arrived at, but analytic techniques allow us to determine that in actuality the processing that the person describes (presumably in good conscience) was not actually employed.

Even people in highly rational enterprises who make decisions of great importance to them (such as investment counselors advising a client on the appropriate make up of a stock portfolio or university faculty members choosing which graduate students to admit) are often unaware of the bases for their decisions or, still worse, think they use bases for deciding that they actually do not employ. ([1], p. 313)

In what has become one of the most highly cited articles across the social sciences, the very next year Nisbett and Wilson wrote:

Generally…we will be blind to contextual factors, or at any rate be particularly poor at disentangling the effects of the stimulus from the context in which it was encountered. Contextual cues are not likely to be spontaneously salient when we are asked, or ask ourselves, why we evaluated an object as we did. Any question about an object is likely to focus our attention on the properties of the object itself and to cause us to ignore contextual cues. ([2], p. 252)

Although a considerable amount of research over the intervening years has confirmed the general proposition that people come to decisions and form judgments in response to cues that influence them below conscious awareness [3–5], the overwhelming majority of this work has been confined to laboratory experiments with limited ecological validity (e.g. [6–9]). For instance, in one experiment, college students who were familiar with the Puma brand evaluated Puma sneakers more favorably after being exposed to a series of photographs of dogs (an animal shown to be conceptually related to the Puma brand) ([10], Experiment 4). In other words, though the students presumably didn’t realize it, merely having seen photos of dogs increased participants’ favorability to a brand that was, at least somewhat, related to dogs. Though useful in their ability to confirm the existence of this phenomenon, these past experiments fall short in their ability to inform what people will do in richer settings with many competing cues. As an example of an investigation that *is* rich in such cues, in another, non-experimental setting, American voters were shown to be influenced by the location of their polling place [11]. Specifically—and again, presumably without their awareness—when American voters happened to cast their ballots in schools, they were significantly more likely to support school-funding initiatives as compared to when they happened to cast their ballots at non-school locations (e.g. churches and community centers). Though this finding certainly meets any ecological validity requirements, its non-experimental nature leaves open the possibility of alternative accounts. As another example, this time experimental, shoppers at a wine store were more likely to purchase German made wines when German style music was playing in the store, and were more likely to purchase French made wines when French style music was playing in the store [12]. This was certainly an ecologically valid test of how contextual cues influence judgments and decisions; however, one limitation of this work is that it is unclear if these shoppers generally knew that such a contextual cue, music, should influence their judgments and decisions. That is, it is unclear and, in fact, unlikely that shoppers would readily predict that music would influence their shopping choices. Accordingly, by providing a rich context with an experimental design, the present study demonstrates that even in situations with many competing cues and where contextual cues are well understood to influence judgments, people fail to realize the influence of those cues. In other words, though contextual cues do, in fact, drive the formation of judgments, when asked to identify and articulate the reasons for the formation of those judgments, people apparently fail to recognize that their judgments were influenced by those exact cues. This is considerably different from the notion that contextual cues generally influence judgments. Instead, we demonstrate the more interesting case where people are well aware that certain cues *should* influence their judgments, yet fail to incorporate such information into their actual judgment formation. Moreover, an additional contribution of the present work is that, to our knowledge, there is little or no previous research examining the unconscious influence of contextual cues where the cues are known by people to have an influence, in a retail setting. By demonstrating the impact of contextual cues in a novel, yet critically important setting, the present work further extends our understanding of how and when contextual cues influence judgment formation.

In keeping with frequent calls for psychology, especially social psychology, to generalize its findings by conducting more “real world” studies of its laboratory-derived propositions (e.g. [13], p. 981; [14–15]), this study was conducted with real-world shoppers intercepted in shopping mall corridors of eight shopping malls geographically dispersed across the United States. These shoppers, upon their agreement, were brought into shielded testing facilities within the malls in order to participate in the study. Though the experiment falls short of true “field experiment” status, as it was conducted in a laboratory-like context, as will be evident, the methodology employed is context rich, uses a diverse population, and asks a real and meaningful question. This ecological validity allows for greater understanding of just how important contextual cues are to forming judgments and just how unaware people are that these cues matter. The experiment was conducted for the purpose of being offered as evidence in a matter adjudicated in federal court, and its presentation in court was credited with the jury deciding for the plaintiff. The specific questions tested are (1) do contextual cues influence judgment formation in rich settings containing many contextual cues and, if so, (2) will people be aware that those cues influenced their judgment formation?

## Background

The litigated matter concerned two food companies. One was a giant in the preserved fruit and vegetable business, selling its products in cans placed on non-refrigerated shelves located in the canned goods sections of supermarkets. The other operated a fresh fruit and vegetable business, selling its goods in plastic containers situated on refrigerated shelves or in refrigerated cabinets in the fresh produce section of supermarkets.

There came a time when the preserved foods manufacturer (hereinafter referred to as PFM) began selling the same preserved grapefruit sections in see-through plastic containers suggestive of the thin, see-through plastic containers used for fresh foods sold in delicatessens and fast food markets. Moreover, instead of situating the grapefruit in the canned/jarred goods sections of supermarkets, PFM began placing these products in the fresh produce sections. Although the containers held preserved fruit that required no refrigeration, they were placed on refrigerated shelves or in refrigerated cabinets, as would be necessary for fresh fruit products. As a result of these three factors–fruit placed in thin, see-through plastic containers; placed in the fresh produce section of supermarkets; placed on refrigerated shelves–the fresh foods company (hereinafter referred to as FFco) was concerned that consumers looking at the fruit in the processed fruit containers would be misled into believing these PFM containers held fresh fruit. A study was conducted to test this hypothesis. Due to a protective order issued by the U.S. District Court hearing this matter, the names of the entities and the raw data collected cannot be made public. However, the anonymized data are available to qualified researchers who contact the corresponding author directly.

## Experiment Overview

The experiment was designed to mimic a real world shopping experience where contextual cues are abundant and likely influence judgment formation in rich and interactive ways. In this case, the judgment was whether the participants thought the containers of grapefruit sections they were shown contained fresh or preserved fruit. Though contextual cues are plentiful, the relevant ones in this case were type of packaging (can vs. see-through plastic container), location in the supermarket (placed on a shelf in the canned goods sections vs. on a shelf in the fresh produce section), and the temperature at which the container was stored (room temperature vs. chilled). The last two factors, location and temperature, were not varied independently of one another because the purpose of this experiment was to mimic, as closely as possible, real world conditions. To that end, canned food is seldom, if ever, located in a refrigerated display, and fresh produce is seldom, if ever, located in a room temperature display. Accordingly, the experiment did not involve these two conditions. To that end, there were two main contextual variables: container type (See-Through vs. Can) and Location/Temperature (Fresh Produce Section–Chilled vs. Canned Food Section–Room Temperature). The research questions are thus: (a) are people’s judgments of fresh vs. preserved fruit influenced by these specific contextual cues and (b) if yes, are people able to identify and articulate where those judgments came from? Importantly, we are not interested in testing the relative strength of these cues, as doing so would not allow any generalizability to other contexts. That is, if we found that container type was a stronger cue than location in forming judgments, it would be unclear if this were true for all types of food or true only for canned fruit. Instead, we are interested in the two key conceptual questions outlined above, which should generalize to many contexts. If we find that a) people use contextual cues in a cue-rich environment, b) people are aware that these cues in general should influence judgment formation, but c) fail to realize this when actually forming judgments, then we can draw a reasonable conclusion about how people form judgments in general, and not just in this one context.

## Method

### Participants and Testing Sites

The universe to which the findings were to be projected was defined as males and females, age 18 and older, who said they were likely to buy cut fruit over the coming month, and who were likely over the coming month to shop at a retail outlets selling cut fruit products (specifically, supermarkets, small grocery or convenience stores, or large discounters such as Walmart, Costco, or Sam’s Club). Specifically, participants were asked three sets of questions: 1) their age, 2) whether they planned to shop at Walmart, Costco, Sam’s Club, a small grocery store or convenience store, or a supermarket in the coming month (Yes/No), and 3) whether they were likely to buy any chilled or refrigerated cut fruit in the coming month (Yes/No). Participants were allowed in the study only if they indicated that they were 1) 18 years of age or older, 2) they planned on shopping in one of the listed stores in the coming month, and 3) they planned on purchasing chilled or refrigerated cut fruit in the coming month. As part of the same screening questionnaire, participants also indicated past purchase behavior in a series of food categories and past purchase behavior in a series of purchase locations. Neither of these sets of questions, however, were used to screen out participants but were in place to provide a clearer picture of who the participants were. The study was conducted in shopping malls in eight geographically dispersed cities, two in each of the four U.S. Census Divisions. The Divisions and cities were as follows.

**Table:** 

Census Division	Cities
Northeast	Woodbridge, NJ; Massapequa, NY;
Midwest	Independence, MO; Detroit, MI;
South	Jacksonville, FL; Atlanta, GA;
West	San Diego, CA; Portland, OR.

Under double-blind conditions, professional interviewers intercepted potential respondents in the shopping mall corridors and asked them a number of screening questions to ensure that interviews would be conducted only with individuals who satisfied the universe definition specified above. Those who qualified were told they would receive $3.00 for completing a brief interview; those who agreed to participate were escorted to a shielded testing room within the mall. Age and gender quotas were set to correspond to best estimates of cut-fruit product consumers so that respondents in each of the three conditions were approximately 30% male and 70% female, with approximately 50% ages 18–29, 30% ages 30–39, and 20% ages 40 and older. In all, there were 405 respondents. See Table 1 for a summary of demographic information by experimental condition.

**Table 1:** Sample Information by Group.

**Geographic Location**	**Group 1**	**Group 2**		**Group 3**	**Χ**^**2**^ **Test**^a^
Northeast	25 (24.3%)	23 (23.2%)		50 (24.6%)	Χ^2^ (6) = .13, p > .99
South	26 (25.2%)	26 (26.3%)		52 (25.6%)	
Midwest	27 (26.2%)	25 (25.3%)		51 (25.1%)	
West	25 (24.3%)	25 (25.3%)		50 (24.6%)	
**Gender**	**Group 1**	**Group 2**		**Group 3**	**Χ**^**2**^ **Test**
Male	31 (30.1%)	29 (29.3%)		60 (29.6%)	Χ^2^ (2) = .02, p > .99
Female	72 (69.9%)	70 (70.7%)		143 (70.4%)	
**Age**	**Group 1**	**Group 2**		**Group 3**	**Χ**^**2**^ **Test**
18–29	54 (52.4%)	48 (48.5%)		103 (50.7%)	Χ^2^ (4) **=** .47, p > .97
30–39	27 (26.2%)	30 (30.3%)	58 (28.6%)		
40 and Over	22 (21.4%)	21 (21.2%)	42 (20.7%)		

^a^Tests for balance across groups. Null result implies no significant difference in sample characteristic across groups.

### Ethics Statement

The authors did not consult an ethics committee or institutional review board prior to the start of the research, because the work was done, originally, for non-academic purposes. However, all research complied with ethical standards prescribed by the Declaration of Helsinki and was approved after the fact for use in publication by the institutional review boards of both Carnegie Mellon University and New York University, because the data in question do not contain identifiable information. Both institutional review boards deemed this research to be “exempt” as defined by federal policy 45 CFR 46 101(b) paragraph 4, and, in doing so, waived the need for informed consent. Additionally, testing was done at permanent testing facilities located in each of the malls described above. Testing facilities are routinely used for commercial marketing and advertising research. Potential respondents (Males and Females age 18 and older) were intercepted in the mall corridors and told the following: “Hello, I’m _____ from [xyz] Research Group, a nationwide market research organization. We’re conducting a survey about people’s reactions to certain products, and your opinions are especially important to us. The interview will take only a few minutes and none of your answers will be associated with you.” Individuals were then asked questions regarding their age, whether they shopped at specific types of food retailers, and the types of products they bought at those retailers. Based on their responses, those who qualified were then told, “You qualify for a survey we are conducting. I have only a few more questions, but before I can ask you these questions, I need to show you some items in our interviewing facility in this mall. It will only take a few minutes and as a thank you for helping us out, we will give you $3.00. Please come with me to our interviewing facility nearby in this mall.” If participants refused, the study was terminated. Only individuals who agreed to participate were questioned further. Participants provided oral consent to participate and were free to discontinue participation at any point.

### Procedure

In order to ensure objectivity of data collection, the research was conducted by a third party who was not made aware of the purpose of the research, and the research was conducted under double-blind conditions. That is, the respondents were kept uninformed about the purpose and sponsorship of the study, and both the interviewers and field supervisors who were charged with collecting the data were similarly “blind” with respect to the purpose and sponsorship. When neither the interviewers nor the respondents have such knowledge, the possibility that either some interviewer(s) or some respondent(s) might correctly guess the purpose and/or the sponsor of the investigation is minimized. At no time were either the supervisors or interviewers told that the study might be used for purposes of litigation.

Identifiable information was collected only for the purpose of record keeping by the company hired to execute the research. That is, information such as names and addresses of participants was never associated with the data, nor was it transferred to the researchers upon completion of the research project. Accordingly, anonymity of participants is complete.

After being seated in a testing room, participants were told, “We need to start by having you watch a short video. This video shows what a shopper might see when walking into a supermarket to buy some cut fruit. As you watch the video, please try to imagine that you were that shopper walking into that supermarket.” All participants next watched one of three videos created by a professional videographer. Each video provided a “from the shopper’s eyes view” of what a consumer would see upon entering a large, modern supermarket and walking either to the chilled refrigeration area within the fresh produce section, or to the canned foods aisle. Approximately one minute in length, the videos differed in terms of their last 10 seconds. For participants in Group 1, the video zoomed in and remained focused on the contested grapefruit sections in the thin, flexible see-through plastic container exactly as it was displayed in the chilled refrigeration area within the supermarket’s fresh produce area, with the identical packaging that was used in the store, including all brand, ingredient, and nutritional information. For participants in Group 2, the video zoomed in and remained focused on the identical product in the thin, flexible see-through plastic container, but this time as it was displayed on a shelf in the supermarket’s canned/jarred foods aisle. For participants in Group 3, the video zoomed in and remained focused on a can of red grapefruit sections as it was displayed in the canned/jarred foods aisle of the supermarket, again with all brand, ingredient, and nutritional information. It’s important to note that in all three instances, the product contained *preserved* red grapefruit sections, not fresh grapefruit sections.

Immediately after the video ended, participants were handed an exemplar of the product shown in the video they had just seen. Those in Group 1 were handed a chilled exemplar of PFM’s processed grapefruit sections product in a plastic, see-through container; those in Group 2 were handed the same processed fruit product as a room temperature exemplar; those in Group 3 were handed a room temperature of PFM’s canned grapefruit sections. All participants were asked to examine their respective products as if they were considering whether or not to buy them. The experimental design thus consisted of manipulating the type of container (thin, flexible, see-through plastic vs. can), section of the supermarket (fresh produce vs. canned/jarred aisle), and temperature of product when handed to the respondent (chilled vs. room temperature). To be clear, three conditions varied the three relevant cues: container, location, and temperature. However, because we employed a nested design, we are unable to independently assess each cue’s relative influence on judgment formation. However, the principle interest of this research is not to understand, say, whether container type or location type has a greater influence on judgment formation, but rather to understand whether these incidental cues have any influence on judgment formation and whether people are aware that these cues are influential. To that end, the independence of the cues is less relevant. For a summary of the differences across groups see Table 2.

**Table 2:** Judgments and Coding of Most Common and Most Relevant Open Ended Responses for Reasons That Participants Believed Product Contains Fresh or Preserved Fruit.

**Condition Description**	**Group 1**	**Group 2**	**Group 3**
Container	See-Through	See-Through	Can
Location	Fresh Produce	Canned Food	Canned Food
Temperature	Cold	Room Temp	Room Temp
**Judgment and Reasons for Judgment Coding**^a^	**Group 1**	**Group 2**	**Group 3**
*Cut Fruit Product Contains Fresh Fruit*	*54 (52*.*4%)*	*32 (32*.*3%)*	*42 (20*.*7%)*
Appearance of the Fruit	41 (75.9%)	25 (78.1%)	15 (35.7%)
Packaging (net)	17 (31.5%)	12 (37.5%)	3 (7.1%)
Because it’s in a plastic see-through container	5 (9.3%)	2 (6.3%)	0
In-Store Location (net)	0	2 (2.0%)	0
Because it’s in a canned aisle	0	1 (1.0%)	0
Not refrigerated	0	1 (1.0%)	0
*Cut Fruit Product Contains Preserved Fruit*	*33 (32*.*0%)*	*50 (50*.*5%)*	*122 (60*.*1%)*
Appearance of the Fruit	9 (27.2%)	20 (40.0%)	17 (13.9%)
Packaging (net)	16 (48.5%)	28 (56.0%)	101 (82.8%)
Because it’s canned	0 (0.0%)	0 (0.0%)	100 (82.0%)
In-Store Location (net)	2 (6.1%)	12 (24.0%)	16 (13.1%)
Because it’s not in the fresh fruit section	0	4 (8.0%)	2 (1.6%)
Not refrigerated	0 (0.0%)	3 (6.0%)	5 (4.1%)
*Don’t Know*	*16 (15*.*6%)*	*17 (17*.*2%)*	*39 (19*.*2%)*
**Total**	**103**	**99**	**203**

^a^Reasons for judgment do not sum to 100% because some participants indicated more than one reason for their judgment. Additionally, some participants provided reasons that were completely irrelevant to the research question.

Of note, Group 3 actually comprised two conditions that varied only in regard to the language on the label on the back of the fruit can. The labels were changed partway through the experiment to reflect changes in actual labeling practices, and the sample size for this group was doubled to ensure that this label change did not meaningfully impact results. Specifically, one version of the label included a small print message on the back of the can stating “No preservatives,” whereas the other version did not. The labels were identical in all other respects. As the labels were virtually identical and results across these two conditions did not differ in any meaningful way, the two groups were collapsed into a single group for all analyses. More specifically, splitting these two groups and re-running all of our analyses do not yield any meaningfully different results.

Immediately after the participants indicated they were done examining the product, the interviewer said, “*For* *ALL* *my questions*, *if you don’t know or don’t have an answer*, *please don’t guess*. *Just tell me you “don’t know” or “don’t have an answer” and we’ll go on to the next question*.*”* The interviewer then asked the first substantive question, “*If you can tell*, *does this cut fruit product contain preserved fruit or fresh fruit?* (To counterbalance for potential order effects, half the participants were asked this question with the key phrases provided in the reverse order, “If you can tell, does this cut fruit product contain fresh fruit or preserved fruit?”) This was followed by asking “What, in particular, makes you say that?” and “Can you tell me more about that?” followed by one probe of “Anything else?” The responses were recorded verbatim.

Immediately after answering the “*does this cut fruit product contain preserved fruit or fresh fruit?”* question, all participants were asked several closed-ended questions, each describing a different decision heuristic for determining whether a cut fruit product contains preserved fruit or fresh fruit. As explained by the interviewer:

Each of these cards describes a different type of information that people may use to tell them whether a cut fruit product contains preserved fruit or fresh fruit. As I hand you each one, I’m going to read what’s on it. For each of these types of information, please tell me if it means or suggests to you that a cut fruit product contains preserved fruit or contains fresh fruit. For any of these, if you have no thoughts about it, you can answer don’t know.

Before being handed to participants one at a time, the cards were shuffled so that the questions were asked in random order. There were two versions of each card counterbalanced across respondents, with one version using the phrase “contains preserved fruit or fresh fruit?” and the other half using the phrase “contains fresh fruit or preserved fruit?” These questions were:

*If the cut fruit product is sold* ***in a can***, *does that mean or suggest to you that the product contains preserved fruit or fresh fruit?*

*If the cut fruit product is sold* ***in the same section of the store as whole fruit*, *vegetables and produce***, *does that mean or suggest to you that the product contains preserved fruit or fresh fruit?*

*If the cut fruit product is sold* ***in a package that says “Must be refrigerated*,*”*** *does that mean or suggest to you that the product contains preserved fruit or fresh fruit?*

*If the cut fruit product is sold* ***on a refrigerated shelf or in a refrigerated cabinet***, *does that mean or suggest to you that the product contains preserved fruit or fresh fruit?*

*If the cut fruit product is sold* ***in a clear*, *see-through plastic package with a re-sealable snap-top lid*,** *does that mean or suggest to you that the product contains preserved fruit or fresh fruit?* (This question was asked only of participants in Groups 1 and 2.)

*If the cut fruit product is sold* ***in a thin*, *flexible see-through plastic package with a re-sealable snap-top lid*,** *does that mean or suggest to you that the product contains preserved fruit or fresh fruit?* (Because it was thought that this modified wording more closely corresponded to the legal issue before the court, the remaining 202 participants in Group 3 were asked this question in lieu of the one saying “…in a clear, see-through…”.)

Because they would have informed participants of the study’s purpose, these questions could not have been asked before having the respondents indicate whether the container to which they were exposed contained fresh or preserved fruit. Finally, participants were thanked for their time and received their remuneration.

## Results and Discussion

In all of our analyses, order of question presentation had no meaningful influence on the results, and so we do not further discuss this. Did contextual cues influence participants’ judgments? As can be seen in Table 2, they did. We test this hypothesis by comparing the rate of “fresh fruit” judgments by variable. Our experimental design allows us to test this in two ways. First, a binary logistic regression revealed that of the 202 participants exposed to the grapefruit sections in a See-Through container (Groups 1 and 2), 86 (42.5%) believed the fruit to be fresh, as compared to only 42 of the 203 participants (20.7%) exposed to the grapefruit sections in a Can container (Group 3; *B* = 1.04, *SE* = .22, *e*^*B*^ = 2.84, *Wald* = 21.70, *p* < .001). When the grapefruit sections were in a see-through container, participants were significantly more likely to believe the fruit was fresh than when the grapefruit sections came in a can. In the previous analysis we pooled Groups 1 and 2, as they both shared the same container type (See-Through). However, a more conservative test is to compare only Group 2 to Group 3, since both had the same Location and Temperature, and varied only in container type. Doing so, a similar binary logistic regression reveals that of the 99 participants in Group 2 (See-Through container), 32 (32.3%) believed the fruit to be fresh compared to only 42 of the 203 participants in Group 3 (Canned Container; 20.7%, *B* = .61, SE = .28, *e*^*B*^ = 1.83, *Wald* = 4.80, *p* = .028). That is, once again, participants believed that the See-Through container was more likely to contain fresh fruit than the Canned container, even though both contained preserved fruit.

Next, we turn to the other two cues: Location and Temperature. As aforementioned, because these two cues were perfectly confounded with each other, we treat them as a single cue: Fresh Produce Section in a Cold container (Group 1) and Canned Food Section in a Room Temperature Container (Groups 2 and 3). Doing so, a binary logistic regression reveals that of the 103 participants shown the product in a Chilled container displayed in the Fresh Produce section (Group 1), 54 (52.4%) believed the fruit sections to be fresh. This compares to only 74 of the 302 participants (25.5%) given to understand that the product came from the Canned Food section or that it was sold at room temperature (Groups 2 and 3; *B* = -1.22, SE = .24, *e*^*B*^ = .30, *Wald* = 26.30, *p* < .001). In other words, when the grapefruit sections were sold in the fresh produce section of a grocery store or were refrigerated, participants were more likely to believe the product contained fresh fruit than when it was sold in the canned food section or sold at room temperature. Of note, even if we compare Group 1 only to Group 2, where the container type is identical, (52.4% vs. 32.3%; *B* = -.84, SE = .29, *e*^*B*^ = .43, *Wald* = 8.22, *p* < .005) or Group 1 only to Group 3, where container type differs, (52.4% vs. 20.7%; *B* = -1.44, SE = .26, *e*^*B*^ = .24, *Wald* = 30.11, *p* < .001), we see the same basic result: participants believe the fruit displayed in the Fresh Produce Section and Chilled is more likely to be fresh, even though it is always preserved.

Not surprisingly, these results demonstrate that people use contextual cues when forming judgments, even when those contextual cues sometimes lead them astray. Although the fruit in all the containers presented to the participants was preserved, depending on condition, participants were more or less likely to believe the fruit was fresh rather than preserved. The more interesting question is whether participants had insight as to the source of their judgments. Assessing this question requires first determining if participants have a reasonable lay understanding of what these various contextual cues generally signal. It would be one thing for participants to be influenced by cues of which they had no understanding; it would be quite another for them to be influenced by cues they actually knew and understood, but then be unable to identify and articulate the presence of these cues.

Do participants know the extent to which the judgment in question (fresh vs. preserved) correlates with these cues? In short, they do. As can be seen in Table 3, in general, participants have lay beliefs that cut fruit products sold in the same section as fresh produce (including whole fruits) or provided in chilled or see-through containers tend to be fresh. Participants’ judgments about fruit freshness derive from their lay beliefs about what the relevant contextual cues signal about fruit freshness in general.

**Table 3:** General Inferences About Contextual Cues.

	Number of participants responding cue indicates product contains fresh fruit			
If cut fruit product is sold…,	Group 1	Group 2	Group 3	Total
…in a can	12 (11.7%)	14 (14.1%)	16 (7.9%)	42 (10.4%)
…in the same section of the store as whole fruit, vegetables and produce	73 (70.9%)	76 (76.8%)	131 (64.5%)	280 (69.1%)
…in a package that says “Must be refrigerated”	68 (66.0%)	59 (59.6%)	130 (64.0%)	257 (63.5%)
…on a refrigerated shelf or in a refrigerated cabinet	67 (65.0%)	64 (64.6%)	147 (72.4%)	278 (68.6%)
…in a clear, see-through plastic package with a re-sealable snap-top lid	60 (58.3%)	46 (46.5%)	NA	106 (52.4%)^a^
…in a thin, flexible see-through plastic package with a re-sealable snap-top lid	NA	NA	125 (61.6%)	125 (61.6%)^b^
N	103	99	203	405

^a^Total based on the 202 participants in Groups 1 and 2

^b^Total based on the 203 participants in Group 3

Having established that people are generally aware that these contextual cues have meaning, we turn to the more interesting question of whether the participants were aware that these cues influenced their judgments about the grapefruit slices. Immediately after answering the first question that asked whether the fruit in the container they were shown was fresh or preserved, participants were asked “What, in particular, makes you say that?” and “Can you tell me more about that?” followed by one probe of “Anything else?” As with open-ended questions of this sort, many answers were given to these three questions. The responses were categorized into approximately 200 classifications, which were rolled up into three general categories (appearance, packaging, and in-store location; see Table 2). A complete set of categories and response frequencies by condition can be obtained from the author upon request. Coding of open-ended responses into these categories was completed by two experienced coders operating independently and blind to the hypotheses being tested. These coders were employees of the subcontractor, a major market research company that implemented the study. The coders’ work was reviewed and accepted by the senior author.

Despite (a) being significantly influenced by the contextual cues in question (Table 2) and (b) knowing that these cues, in general, have some evidentiary value (Table 3), participants overwhelmingly were unable to identify and/or articulate that these cues had any influence over their judgment formation. Of the 128 participants across the three conditions who reported that the fruit was fresh, 81 (63.2%) indicated that the reason for their judgment was the appearance of the fruit (e.g. “Because it looks like fresh fruit,” “I think it looks fresh,” and “It looked juicy and good”). In contrast, only 32 (25.0%) indicated that the reason for their judgment was the packaging (e.g., “The look of the fruit through the clear container,” “The way it's packaged,” and “The container it's in”), and only 2 (2.0%) indicated that the reason for their judgment was the location within the store (e.g., “Where it was located in the store”). Thus, even though the contextual cues exerted a significant influence over their judgments of freshness, participants seemed unable to identify and articulate that those cues had any influence at all. This was true despite the fact that, in general, participants were well aware that those same contextual cues do correlate with fruit freshness.

Though the failure to appreciate the source of their judgments was overwhelmingly apparent for participants who incorrectly believed the fruit to be fresh, participants who believed the fruit to be preserved varied in the degree to which they appreciated the influence of the contextual cues. Specifically, participants tested on the canned container (Group 3) who indicated the fruit was preserved were quite accurate in translating the general belief that canned fruit is preserved (Table 3) into the reason for their specific judgment. Indeed, 101 (82.8%) of the 122 participants in Group 3 who indicated that the fruit was preserved correctly attributed the reason for their judgment to the fact that the fruit came in a can. This is compared to only 44 (53.0%; *B* = -1.45, SE = .33, *e*^*B*^ = .24, *Wald* = 19.86, *p* < .001) of the 83 participants in Groups 1 and 2 who indicated that the reason they believed the fruit was preserved was because it came in a see-through container (something that is inconsistent with their stated beliefs about fruit freshness). These findings are consistent with Nisbett and Wilson’s observation that “Any question about an object is likely to focus our attention on the properties of the object itself and to cause us to ignore contextual cues” ([2], p. 252). Participants tested on the see-through plastic container could see the object (fruit) inside and thus explained their judgment in terms of the fruit, not the contextual cues. In contrast, participants tested on the can could not see the object (fruit) inside and thus were more likely to explain their judgment in terms of the dominant contextual factor (the can), not the fruit inside.

## Conclusion

This study tested a key proposition advanced by Nisbett and Wilson [2] that had heretofore been primarily supported by laboratory research: namely, that people’s judgments are greatly influenced by contextual cues, even though they often are unaware of these cues having influenced them at all. By replicating these findings in an ecologically rich and complex environment, this research contributes to their robustness and generalizability. Robustness and generalizability is further enhanced by the fact that the participants in the experiment came from highly diverse backgrounds (both geographically and demographically) and the setting studied was a close approximation of the real world that people regularly experience. This study also adds a perplexing wrinkle that remains to be explained. It is one thing for people to be influenced by cues about which they have no understanding; it is quite another for them to be influenced by cues they actually know and seem to readily understand, but then be unable to identify and articulate the presence or operation of these cues. This issue is left for future research.

One question still unanswered is if these shifts in preferences influence subsequent behavior. That is, though we demonstrate that contextual cues influence judgments about the nature of the products being evaluated, it is not clear if these differing judgments will influence subsequent purchase behavior. For instance, if people generally believe that fresh fruit is of higher quality than preserved fruit, it follows, all else being equal, that people would be more willing to purchase fruit they believe to be fresh. Though we never directly address this question, ample work shows that attitudes towards products predict subsequent behavior quite well. For instance, following a trial of a snack food, participants’ evaluations of that food strongly predicted their likelihood to subsequently purchase it [16]. For a more thorough discussion of how attitudes predict behavior, see Fazio 1990 [17].

Our experimental design was framed around feasible considerations for a manufacturer of preserved grapefruit slices. That is, we utilized only three experimental conditions, which forced us to confound some of the contextual cues. This was done largely because it would be unreasonable to expect cold products to be displayed in a canned food section (since refrigeration units are never placed in such a location in a store), and thus the cold products were always in the fresh food section with refrigeration and canned food products were always in the canned food section without refrigeration. Because we confounded temperature and location, we were unable to test the relative influence of these contextual cues on judgments of the preserved vs. fresh nature of the fruit. However, this limitation does not speak at all to the general conclusion that contextual cues influence judgments, often without people even being aware of such influences. That is, we are not, and should not be, interested in temperature per se, but are, rather, interested in understanding if contextual cues influence judgments without people’s awareness. To that end, our current design accomplishes this task. Had we chosen to un-confound these two factors, we certainly would have been able to learn the relative strength of these cues in this particular context, but doing so would add little to the question of primary interest.

A final consideration of the present work is what boundary conditions exist both in terms of when contexts influence individuals and when individuals fail to realize that the context is influencing them. For instance, it is possible that shoppers highly engaged with the product are less likely to be susceptible to such context effects because they have much more accurate information about the preserved nature of fruit based on repeated previous exposures to the product. Likewise, when the general context is less complex than the mosaic that is the supermarket, it is possible that incidental cues such as product packaging may be less influential in forming judgments. Finally, to the question of when individuals are likely to actually be aware of when contextual cues are influencing them, consumers who are particularly thoughtful when forming judgments about preserved vs. fresh fruit are more likely to be aware of the environment in which their judgments are being formed. That is, to the extent that someone takes great care in thinking about why a cup of fruit may be fresh or preserved, they may be more likely to be sensitive to otherwise unnoticed contextual factors. Additionally, when the contextual factors are made particularly salient, as is the case in joint-evaluations [18], people are far more likely to realize that context influences their judgments. In other words, if consumers were to notice the same container of fruit both in the fresh fruit section and the canned goods section of a supermarket, they would be far more likely to realize that location is a factor that might inform the preserved nature of the fruit. In sum, our experiment shows that the general tendency for consumers is to use contextual cues when forming judgments, but to fail to realize they have done so. However, it is also the case that this need not be true in all circumstances, as outlined above.
